# *Connexin 43* enhances liver metastatic ability of GIST cells *in vivo*

**DOI:** 10.3389/pore.2026.1612383

**Published:** 2026-06-04

**Authors:** Takako Kihara, Jiayin Yuan, Takashi Yamasaki, Makoto Yoshida, Mizuka Ohkouchi, Yuka Nakaya-Hashikura, Neinei Kimura, Kanae Hyodo, Chisato Ohe, Seiichi Hirota

**Affiliations:** 1 Department of Diagnostic Pathology, Hyogo Medical University School of Medicine, Nishinomiya, Hyogo, Japan; 2 Department of Pathology, First People’s Hospital of Foshan, Foshan, Guangdong, China

**Keywords:** *Connexin 43*, gastrointestinal stromal tumor, interaction with vascular endothelial cells, invasion, liver metastasis

## Abstract

**Objective:**

Small intestinal gastrointestinal stromal tumors (SI-GISTs) have a higher risk of distant metastasis and recurrence than gastric GISTs (G-GISTs). However, the underlying mechanisms contributing to the poor prognosis of SI-GISTs remain elusive. Previous research has demonstrated that SI-GISTs exhibit elevated expression of *Connexin 43* (*Cx43*), a component of gap junctions, whereas G-GISTs exhibit minimal expression. The differential expression of *Cx43* between G-GISTs and SI-GISTs may account for their distinct clinical behavior. This study aimed to investigate the impact of *Cx43* on the liver metastatic potential of GIST cells, both *in vivo* and *in vitro*.

**Methods:**

To elucidate the relationship between *Cx43* expression and poor prognosis in SI-GISTs, we conducted a comparative analysis of original GIST-T1 cells, which express minimal levels of *Cx43* and represent G-GISTs, and GIST-T1 cells engineered to express high levels of *Cx43* through transfection with *Cx43* cDNA (GIST-T1-Cx43 cells), representing SI-GISTs. This was achieved using a newly developed *in vivo* liver metastasis xenograft mouse model, and the results were corroborated by conventional *in vitro* experiments.

**Results:**

In GIST cells, *Cx43* enhanced the liver metastatic potential *in vivo* (p = 0.010). *In vitro*, *Cx43* suppressed cell proliferation (p < 0.001) while promoting migration (p < 0.001), invasion (p = 0.036), tumor-endothelial cell adhesion (p < 0.001), and transendothelial migration (p < 0.001).

**Conclusion:**

Elevated *Cx43* expression may contribute to the poor prognosis of patients with SI-GISTs by enhancing their metastatic potential. *Cx43* represents a potential novel therapeutic target for the inhibition of SI-GIST metastasis.

## Introduction

Gastrointestinal stromal tumors (GISTs) are the most prevalent mesenchymal tumors of the gastrointestinal tract [1]. These tumors can manifest throughout the digestive system, with the highest incidence in the stomach (60%–70%), followed by the small intestine (20%–25%) [2]. Given that interstitial cells of Cajal (ICCs), which function as pacemaker cells for gastrointestinal motility [3], express KIT (CD117) similarly to GISTs [4–6], it is now posited that GISTs originate from or differentiate into ICCs [1].

Most sporadic GISTs exhibit gain-of-function mutations in either the *KIT* gene (80%–85%) or the *PDGFRA* gene (5%–10%), which encode the KIT tyrosine kinase and alpha-subunit of the platelet-derived growth factor receptor alpha tyrosine kinase, respectively [1, 7, 8]. The majority of *KIT* mutations in sporadic GISTs are identified in exon 11, followed by exon 9, with a smaller proportion occurring in exon 8, 13, or 17 [9]. Approximately 10% of GISTs are devoid of *KIT* and *PDGFRA* mutations, classifying them as wild-type GISTs [10], which may possess mutations in the *NF1*, *BRAF*, or *SDH* complex genes [11–13].

Small intestinal gastrointestinal stromal tumors (SI-GISTs) exhibit distinct biological characteristics compared to gastric GISTs (G-GISTs). A significant distinction is that nearly all GISTs with *PDGFRA* mutations are located in the stomach, whereas the majority of GISTs with *KIT* exon 9 mutations predominantly occur in the small intestine and are associated with a poor prognosis [14]. GISTs exhibit unique transcriptional profiles that correlate with their *KIT* genotype and anatomical location [15, 16]. SI-GISTs have a higher propensity for metastasis and recurrence and are considered to have a worse prognosis than G-GISTs of equivalent tumor size and mitotic rate [17–19]. Consequently, risk classification for GIST recurrence, which includes the anatomical tumor site as a risk factor, is extensively used in clinical practice.

In our previous study, we observed that the majority of SI-GISTs exhibit high levels of *Connexin 43* (*Cx43*) expression, whereas G-GISTs demonstrate minimal expression of this protein [20]. Cx43 is a four-pass transmembrane protein that facilitates the formation of hemichannels and gap junctions, which are essential for the transmission of ions, small molecules, and metabolites [21, 22]. A hemichannel is composed of six Cx43 molecules, and gap junctions are formed by pairing two hemichannels [22, 23]. Previous studies have indicated that *Cx43* expression varies among different tumors and is influenced by the stage of tumor progression [21, 22]. In colorectal cancer [24] and pancreatic cancer [25], *Cx43* acts as a tumor suppressor. Conversely, in breast cancer [26], gastric cancer [27], melanoma [28], and ovarian cancer [29], *Cx43* is considered to act as a tumor promoter, as metastatic lesions exhibit elevated *Cx43* expression at both the protein and mRNA levels compared to primary lesions.

In the context of Cx43 immunohistochemical staining for breast cancer, similar to the findings in normal mammary glands and benign lesions, Cx43 immunostaining is absent in non-invasive ductal carcinoma. In contrast, in invasive ductal carcinoma, Cx43 is predominantly found in the cytoplasm, with occasional punctate staining of the membrane [22, 30]. Furthermore, compared to the primary site, there is an increase in *Cx43* expression in lymph node metastases, marked by intensified cytoplasmic and membrane staining of Cx43 in the metastatic foci [22, 26]. A recent study has indicated that *Cx43* functions as a tumor suppressor in colorectal cancer when located on the cell membrane, but its translocation to the nucleus is linked to the progression and metastasis of colorectal cancer [31].

In this study, we examined the liver metastatic potential of GIST-T1 cells with elevated *Cx43* expression, achieved through transfection with *Cx43* cDNA (GIST-T1-Cx43 cells), in comparison to the original GIST-T1 cells with lower *Cx43* expression, *in vivo*. Additionally, we assessed the impact of *Cx43* on GIST cells concerning cell proliferation, migration, invasion, and interaction with vascular endothelial cells *in vitro*. Our *in vivo* findings indicate that *Cx43* enhances liver metastasis. *In vitro*, *Cx43* was found to suppress cell proliferation while promoting migration, invasion, tumor-endothelial cell adhesion, and transendothelial migration, providing evidence that *Cx43* augments metastatic potential. *Cx43* may play a crucial role in the metastasis of SI-GISTs and could serve as a potential target for inhibiting GIST metastasis.

## Materials and methods

### Cell lines and mice

The GIST-T1 cell line was derived from a metastatic pleural tumor originating from a G-GIST in a 47-year-old Japanese woman with a heterozygous mutation in *KIT* exon 11 (p.V560_Y578del). This cell line was procured from Cosmo Bio (Cosmo Bio Co., Ltd., Tokyo, Japan) and cultured in Dulbecco’s modified Eagle’s medium (DMEM) (Sigma-Aldrich; Merck KGaA, Darmstadt, Germany) supplemented with 10% fetal bovine serum (FBS) (Biowest, Nuaillé, France), 100 U/mL penicillin G, and 100 μg/mL streptomycin (Invitrogen; Thermo Fisher Scientific, Waltham, MA, USA) at 37 °C in a 5% CO_2_ atmosphere. Human umbilical vein endothelial cells (HUVECs), sourced from the endothelium of veins in the human umbilical cord, were obtained from Takara Bio (Takara Bio, Inc., Shiga, Japan) and maintained in Endothelial Cell Growth Medium 2 (Takara Bio, Inc.).

Female NOD.Cg-PrkdcscidIl2rgtm1Sug/ShiJic (NOG) mice, aged 7 weeks and sourced from Jackson Laboratory Japan, Inc., Japan, with an average body weight of 17–20 g, were utilized and allocated into two distinct groups. The mice were housed in cages with three or four mice per cage, provided with unrestricted access to food and water, and maintained under a 12-hour light and 12-hour dark (12:12 LD) cycle.

### Plasmid construction and transfection

Plasmid construction and transfection were performed as previously described [32]. Full-length *Cx43* cDNA was synthesized via reverse transcriptase polymerase chain reaction (RT-PCR) using the forward and reverse primers specified in Supplementary Table S1, Ampli Taq Gold (Thermo Fisher Scientific), and mRNA extracted from a SI-GIST exhibiting high *Cx43* mRNA expression. The amplified DNA fragments were subjected to electrophoresis, collected, digested with restriction enzymes, and subcloned into the BamHI and XhoI sites of the pcDNA3.1/Zeo (+) mammalian expression vector with the CMV promoter (Thermo Fisher Scientific). To confirm the authenticity of the product, the vector containing the full-length *Cx43* cDNA was sequenced using ABI BigDye Terminator version 3.1 (Applied Biosystems; Thermo Fisher Scientific) and ABI Prism 3100Avant Genetic Analyzer (Applied Biosystems; Thermo Fisher Scientific). A total of 1 × 10^6^ GIST-T1 cells were suspended in 2 µg of the vector with full-length *Cx43* cDNA in 100 µL of Cell Line Nucleofector kit V solution (Lonza, Basel, Switzerland) and electroporated using an Amaxa Nucleofector II machine (program T20) (Lonza) in accordance with the manufacturer’s protocol. Stable GIST-T1 cells with elevated *Cx43* expression (GIST-T1-Cx43 cells) were selected by incrementally increasing the concentration of Zeocin (Thermo Fisher Scientific) to 250 μg/mL, and a single monoclonal clone was isolated using the limiting dilution method. Cloning was performed twice. Western blot analysis was performed as described previously [32, 33]. Equivalent amounts of protein lysates from the two GIST cell lines were applied to Bolt 4%–12%, Bis-Tris Plus WedgeWell^TM^ Gels, electrophoresed, and transferred to an iBlot 2 PVDF transfer membrane (Invitrogen, CA, USA). Polyclonal rabbit anti-Connexin 43 antibody (Sigma-Aldrich; Merck KGaA, C6219, dilution 1:2000), polyclonal rabbit anti-human CD117, c-kit antibody (DAKO Cytomation, Glostrup, Denmark, A4502, dilution 1:500), and monoclonal mouse anti-beta actin antibody (Abcam, Cambridge, UK, ab8226, dilution 1:1000) were used as primary antibodies, and HRP-conjugated goat anti-rabbit IgG antibody (DAKO Cytomation, dilution 1:2000) and HRP-conjugated goat anti-mouse IgG antibody (DAKO Cytomation, dilution 1:1000) were used as secondary antibodies. The membranes were incubated with primary and secondary antibodies using the iBind^TM^ Solution Kit (Invitrogen). Protein bands were enhanced using Amersham ECL Prime Western blotting Detection Reagent (GE Healthcare Life Science, Buckinghamshire, UK) and visualized using a ChemiDoc Imaging System (Bio-Rad Laboratories, CA, USA).

### Liver metastasis xenograft mouse models

**FIGURE 1:**
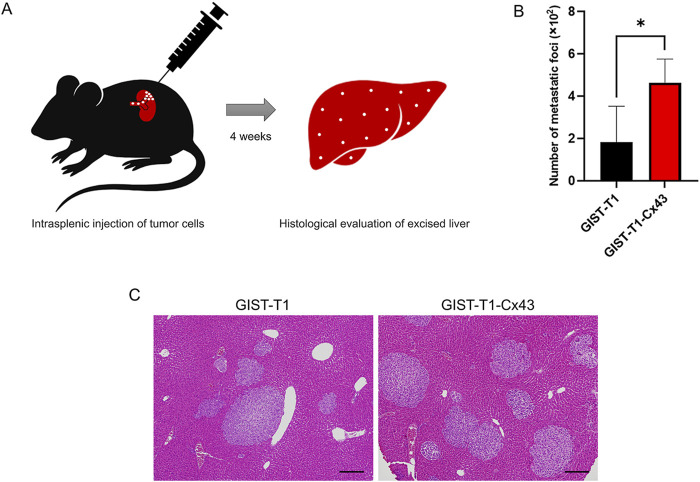
Enhancement effect of *Cx43* expression on metastatic potential of GIST-T1 cells *in vivo*. **(A)** Experimental design of *in vivo* liver metastasis mouse models generated by intrasplenic injection of tumor cells is shown. **(B)** The number of liver metastatic foci in intrasplenic injection models of GIST-T1-Cx43 cells was significantly larger than the number of liver metastasis in intrasplenic injection models of GIST-T1 cells. The data are expressed as the mean ± SD (n = 5–6/each group). *p < 0.05. **(C)** Representative microscopic images of liver metastatic foci in intrasplenic injection models of GIST-T1 cells and GIST-T1-Cx43 cells are shown. Original magnification ×100. Scale bar = 200 μm.

Thirteen female NOG mice, aged 7 weeks, were allocated to two groups. Anesthesia was administered using isoflurane at a concentration of 4%–5% for induction and 1.5%–3% for maintenance. The peritoneum was excised to a length of approximately 15 mm to expose the spleen. Two suspensions of GIST cell lines in DMEM supplemented with 10% FBS (2 × 10^6^ cells/100 µL) were meticulously injected into the spleen using a 29-G needle. After confirming the absence of bleeding at the injection site, the spleen was repositioned within the abdominal cavity, and the peritoneum and skin were sutured with stainless-steel wound clips. To ensure stable cell transplantation, the spleen was retained in the abdominal cavity without dissection. Four weeks after the intrasplenic injection of tumor cells, the mice livers were carefully excised, and the number of liver metastases was analyzed histologically (Figure 1A).

### Histological evaluation of liver metastases

Dissected liver tissues from NOG mice with intrasplenic transplantation of the two GIST cell lines were fixed in 10% neutral-buffered formalin. The tissues were cross-sectioned at 2 mm intervals, dehydrated, and embedded in paraffin. Three-micrometer-thick sections were cut and used for conventional hematoxylin and eosin (H&E) staining. The number of liver metastases in all H&E-stained specimens was evaluated histologically. Three or more tumor cells were counted as one metastatic focus in the liver.

### Cell proliferation assay

Two GIST cell lines were seeded into six wells per cell line at a density of 1 × 10^3^ cells/well in DMEM supplemented with 10% FBS in 96‐well plates (Corning, Inc., NY, USA). Following a 7-day incubation period, 10 μL of Cell Counting Kit-8 (Dōjindo Laboratories, Kumamoto, Japan) was added to each well, and the plates were incubated at 37 °C for an additional 2 h, according to the manufacturer’s protocol. Cell viability was assessed using a microplate reader (2030 ARVO X4, PerkinElmer Life and Analytical Sciences, Shelton, USA). The experiments were conducted in triplicate.

### Migration and invasion assays

As previously described with certain enhancements [32], a migration assay was conducted using Matrigel-free Falcon cell culture inserts (Corning, Inc.), while an invasion assay was performed using 24-well BD BioCoat Matrigel Invasion Chambers (BD Biosciences, NJ, USA), according to the manufacturer’s protocol. Given the stability of the results, two GIST cell lines were suspended at a concentration of 2 × 10^6^ cells/mL in DMEM supplemented with 0.4% FBS and introduced into the upper chambers of the inserts, followed by incubation for 48 h. The cells that migrated, invaded, and adhered to the bottom surface of the membranes were permeabilized in 100% methanol, fixed in 10% neutral buffered formalin, and stained with Giemsa stain for 1 h at room temperature. The total number of migrated and invaded cells on the membrane was quantified using a Hybrid Cell Count BZ-X710 system (Keyence, Corp., Osaka, Japan). The experiments were conducted in triplicate.

### Tumor-endothelial cell adhesion assay

To investigate the interactions between tumor cells and HUVECs, a static adhesion assay was conducted as previously described, with some modifications [32]. A migration assay was performed using Matrigel-free Falcon cell culture inserts (Corning, Inc.), and an invasion assay was performed using 24-well BD BioCoat Matrigel Invasion Chambers (BD Biosciences, NJ, USA) according to the manufacturer’s protocol [32]. HUVECs were cultured at a density of 2.5 × 10^5^ cells/well in 96-well plates overnight. Two GIST cell lines were labeled with 2 μg/mL Calcein-AM (Dōjindo Laboratories) at 37 °C for 30 min, washed thrice with phosphate-buffered saline (PBS), and suspended at a density of 2 × 10^6^ cells/mL in DMEM supplemented with 0.4% FBS to ensure stable results. The two GIST cell lines were seeded at a density of 5 × 10^5^ cells/well on confluent HUVEC monolayers. Following a 2-h co-culture period, non-adherent tumor cells were removed along with the medium, and tumor cells adhered to HUVECs were washed three times with PBS along with HUVECs. Fluorescence intensity was measured using a fluorescence microplate reader (2030 ARVO X4, PerkinElmer Life and Analytical Sciences) at excitation and emission wavelengths of 485 and 535 nm. The experiments were conducted in triplicate.

### Transendothelial migraion assay

To evaluate the transmigration of tumor cells through the endothelial monolayer, a transendothelial migration assay was performed as previously described, with some modifications [32]. HUVECs were pre-seeded at a density of 2 × 10^5^ cells per well onto 24-well Transwell Inserts (Corning, Inc.) and cultured until a monolayer was established. Subsequently, 5 × 10^4^ gastrointestinal stromal tumor (GIST) cells labeled with 2 μg/mL CalceinAM were introduced into the upper chamber of the insert. Following a 48-h co-culture period, non-migrated cells on the upper side of the membrane were removed using a cotton swab. Transmigrated cells on the lower side of the membrane were fixed with 10% neutral-buffered formalin. Transmigrated cells were visualized and quantified in five random fields at ×100 magnification using a fluorescence microscope equipped with a Hybrid Cell Count BZ-X710 system (Keyence, Corp.). The experiments were conducted in triplicate.

### Western blot analysis

To explore the impact of *Cx43* overexpression on the downstream signaling pathways of KIT in GIST cells, Western blot analysis was performed using the same method used to confirm the stable cell line. After assessing the protein concentrations in the lysates from the two GIST cell lines, equivalent amounts of protein from each sample were electrophoresed. Mouse anti-phospho-KIT (pTyr936) antibody (Affinity BioReagents, Inc., Golden, USA, dilution 1:1000), polyclonal rabbit anti-human CD117, c-kit antibody (DAKO Cytomation, A4502, dilution 1:500), phospho-p44/42 MAPK (Erk1/2) (Thr202/Tyr204) antibody (Cell Signaling Technology, Inc., Beverly, MA, USA, 9101, dilution 1:1000), p44/42 MAPK (Erk1/2) antibody (Cell Signaling Technology, Inc., 9102, dilution 1:1000), phospho-Akt (Ser473) antibody (Cell Signaling Technology, Inc., 9271, dilution 1:1000), Akt antibody (Cell Signaling Technology, Inc., 9272, dilution 1:1000), and monoclonal mouse anti-beta actin antibody (Abcam, ab8226, dilution 1:1000) were used as primary antibodies, and HRP-conjugated goat anti-rabbit IgG antibody (DAKO Cytomation, dilution 1:2000) and HRP-conjugated goat anti-mouse IgG antibody (DAKO Cytomation, dilution 1:1000) were used as secondary antibodies.

### Statistical analysis

To compare the number of liver metastases in the two groups of mice transplanted with GIST cell lines in the *in vivo* experiment, the mean and standard deviation (SD) for each group were calculated. The significance of the differences (p < 0.05) between groups was assessed using a two-tailed t-test with Welch’s correction. Grubbs’ test (α = 0.1) was used to identify and statistically exclude a single outlier in each group after confirming data normality. The significance of the *in vitro* experiments was evaluated using an unpaired Student’s t-test. All statistical analyses were performed using GraphPad Prism 10.6.0 (GraphPad Software, Inc., Boston, MA, USA).

## Results

### Western blot analysis of Cx43 protein expression in GIST-T1 cells and GIST-T1-Cx43 cells

**FIGURE 2:**
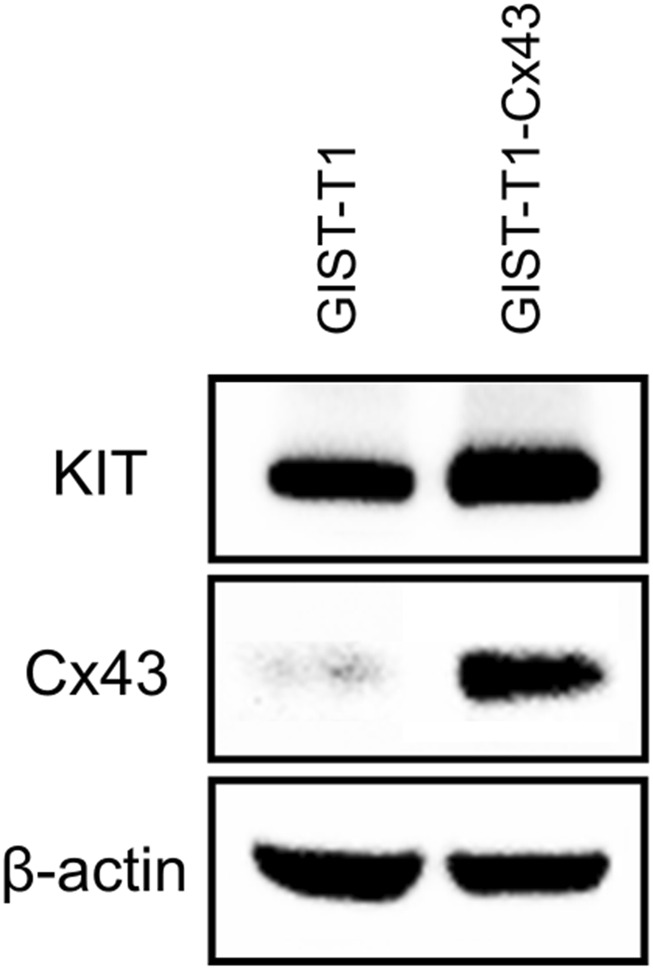
Western blot analysis of Cx43 protein expression in GIST and GIST-T1-Cx43 cells. Western blot analysis showed that Cx43 expression was higher in GIST-T1-Cx43 cells than in original GIST-T1 cells.

As previously reported, Cx43 protein expression was absent in G-GISTs [20]. Western blot analysis indicated that Cx43 protein expression in GIST-T1-Cx43 cells was significantly higher than that in the original GIST-T1 cells (Figure 2). These cells were established as stable GIST-T1 cells expressing elevated levels of *Cx43*, which was achieved by transfecting full-length *Cx43* cDNA into the GIST-T1 cells.

### Enhancement effect of *Cx43* expression on liver metastatic ability of GIST cells

To investigate the impact of *Cx43* on the hepatic metastatic potential of GIST cells *in vivo*, an intrasplenic transplantation experiment was performed in mice. Four weeks after the injection of tumor cells into the spleens of 13 mice, the number of liver metastases was assessed using H&E-stained specimens. The number of hepatic metastatic foci in mice transplanted with GIST-T1-Cx43 cells was significantly 2.53 times greater than that in mice transplanted with GIST-T1 cells (p = 0.010) (Figures 1B,C). A single outlier in each group was statistically identified and excluded following confirmation of data normality (Supplementary Figures S1, S2).

### Suppressing effect of *Cx43* expression on cell proliferation and promoting effect of *Cx43* expression on migration and invasion of GIST cells

**FIGURE 3:**
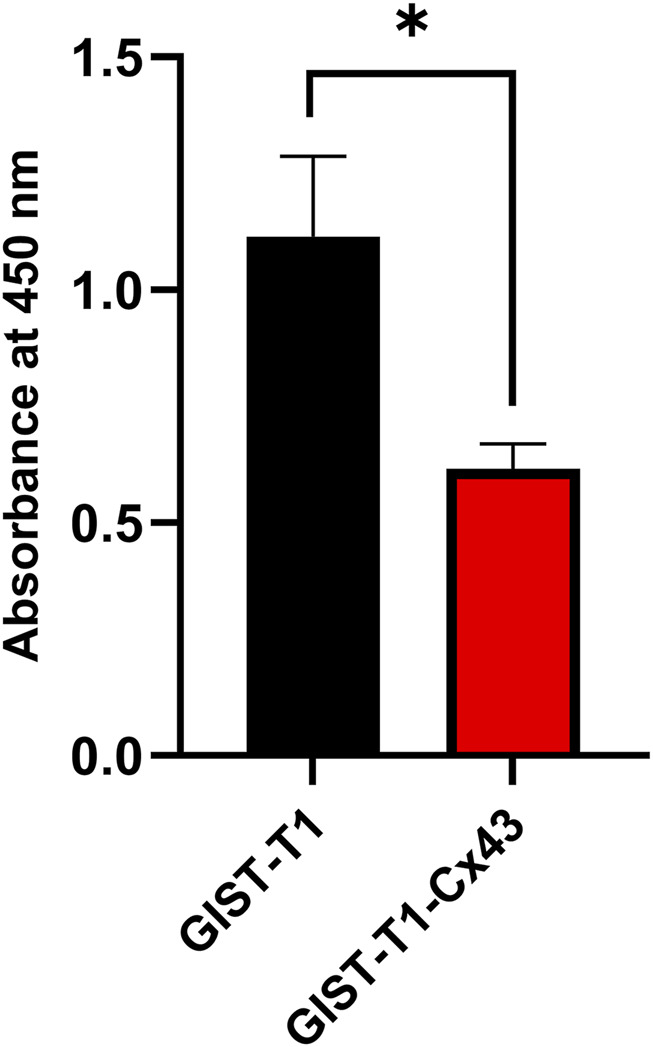
Inhibitory effect of *Cx43* expression on GIST-T1 cell growth *in vitro*. The cells (1 × 10^3^ cells/well) were seeded at in 96-well plates and the number of cells was counted at day 7. After day 7, the number of GIST-T1-Cx43 cells was significantly less than the number of GIST-T1 cells. The data are expressed as the mean ± SD (n = 6/each group). *p < 0.05.

**FIGURE 4:**
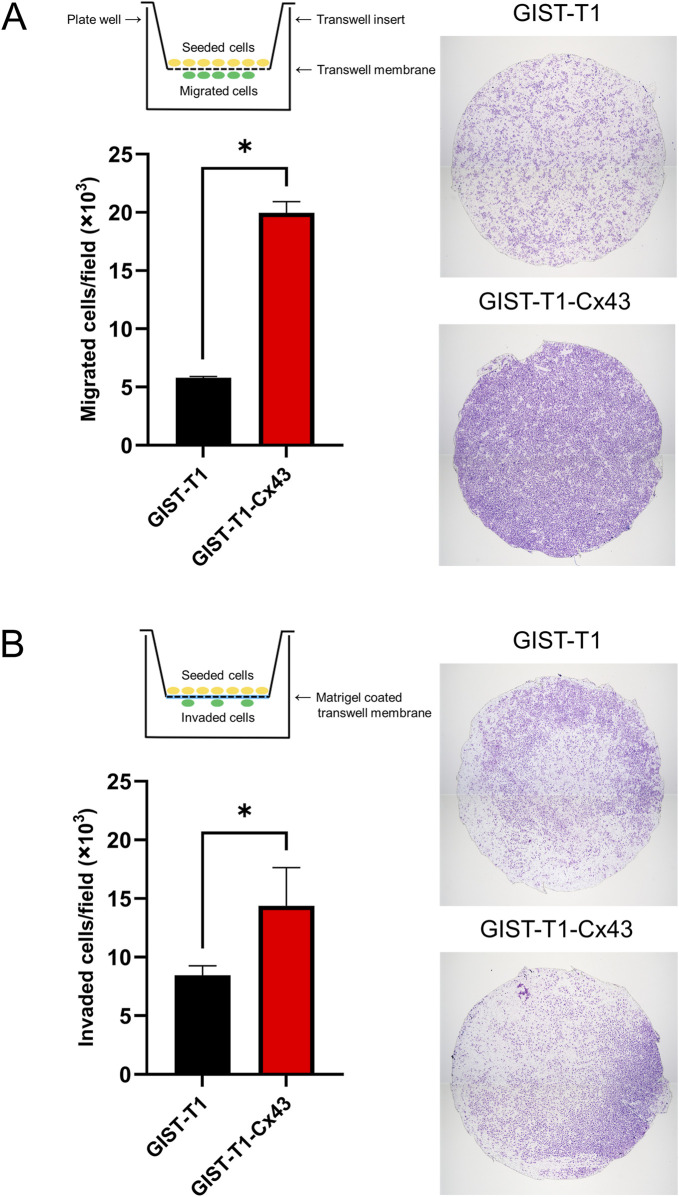
Promoting effect of *Cx43* expression on GIST-T1 cell migration and invasion *in vitro*. The means of microscopic field per membrane in the cell **(A)** migration and **(B)** Matrigel invasion assays were calculated. (Upper left) Schematic diagram of transwell migration and invasion assay is shown. (Lower left) There were more migrated and invaded GIST-T1-Cx43 cells than migrated and invaded original GIST-T1 cells. Right: Representative images of cell migration and Matrigel invasion in Transwell assays using original GIST-T1 cells and GIST-T1-Cx43 cells. The data are expressed as the mean ± SD (n = 3/each group). *p < 0.05.

To assess the influence of *Cx43* on GIST cell proliferation, we conducted a comparative analysis of the proliferative capacities of the two GIST cell lines. Following the seeding of 1 × 10^3^ cells from each cell line and a 7-day incubation period, GIST-T1-Cx43 cells exhibited significantly reduced growth compared to GIST-T1 cells (p < 0.001) (Figure 3). Additionally, we evaluated the impact of *Cx43* on the migratory and invasive behaviors of GIST cells using Matrigel-free and Matrigel-coated transwell membranes, respectively. The GIST-T1-Cx43 cells demonstrated a 3.43-fold increase in migration through the transwell membrane compared to GIST-T1 cells (p < 0.001) (Figure 4A), and a 1.70-fold increase in invasion through the Matrigel-coated transwell membrane (p = 0.036) (Figure 4B).

### Augmentative effect of *Cx43* expression on adherence to HUVECs and transmigration of GIST cells through HUVECs

**FIGURE 5:**
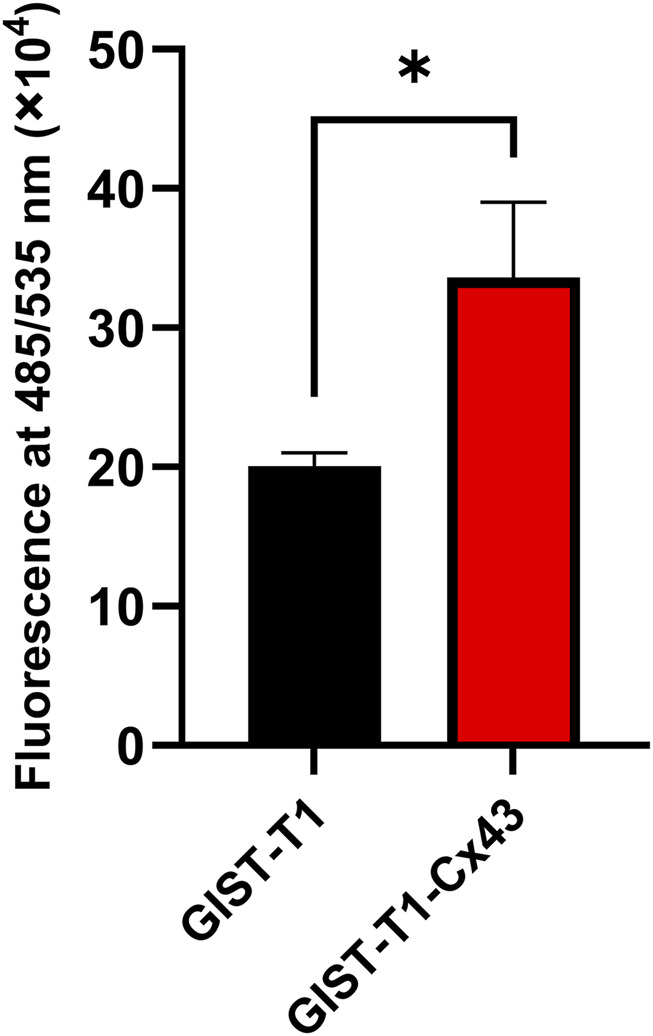
Promoting effect of *Cx43* expression on adhesion of GIST-T1 cells to endothelial cells. Significantly greater numbers of Calcein-AM labeled GIST-T1-Cx43 cells than Calcein-AM labeled original GIST-T1 cells adhered to HUVECs. The data are expressed as the mean ± SD (n = 6/each group). *p < 0.05.

**FIGURE 6:**
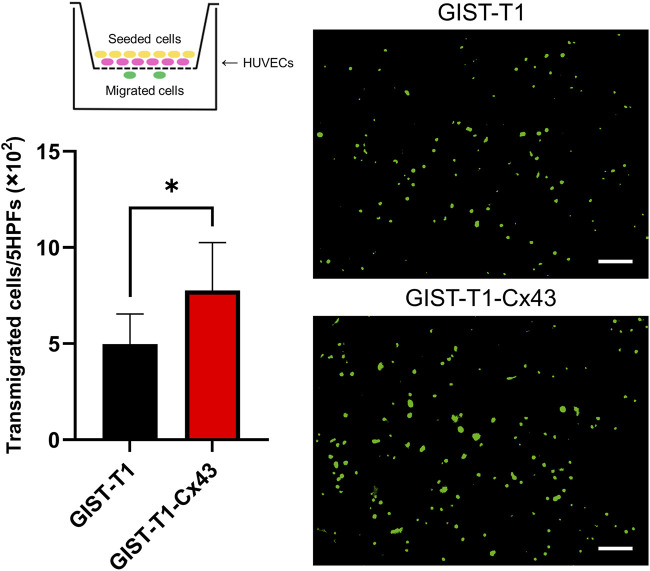
Promotirng effect of *Cx43* expression on transendothelial migration of GIST-T1 cells. (Upper left) Schematic diagram of transendothelial migration assay is shown. (Lower left) The mean numbers of the two GIST cells in 5 random fluorescence microscopic fields per membrane under ×100 magnification are shown. The number of GIST-T1-Cx43 cells showing transendothelial migration was significantly higher than that of GIST-T1 cells showing transendothelial migration. The data are expressed as the mean ± SD (n = 3/each group). *p < 0.05. (Right) Representative images of transmigrated GIST-T1 cells and GIST-T1-Cx43 cells labeled by Calcein-AM green dye are shown. Original magnification ×400. Scale bar = 50 μm.

To assess the influence of *Cx43* on the adhesion of GIST cells to HUVECs, a static adhesion assay was performed. The results indicated that CalceinAM-labeled GIST-T1-Cx43 cells exhibited significantly higher adhesion to HUVECs, with a 1.67-fold increase compared to CalceinAM-labeled GIST-T1 cells (p < 0.001) (Figure 5). Furthermore, to evaluate the impact of *Cx43* on the transmigration of GIST cells through HUVECs, a transendothelial migration assay was performed using three distinct cell lines. The findings revealed that CalceinAM-labeled GIST-T1-Cx43 cells migrated through HUVECs 1.56 times more than CalceinAM-labeled GIST-T1 cells (p < 0.001) (Figure 6).

### Activated effect of *Cx43* expression on RAS-RAF-MAPK pathway in GIST cells

**FIGURE 7:**
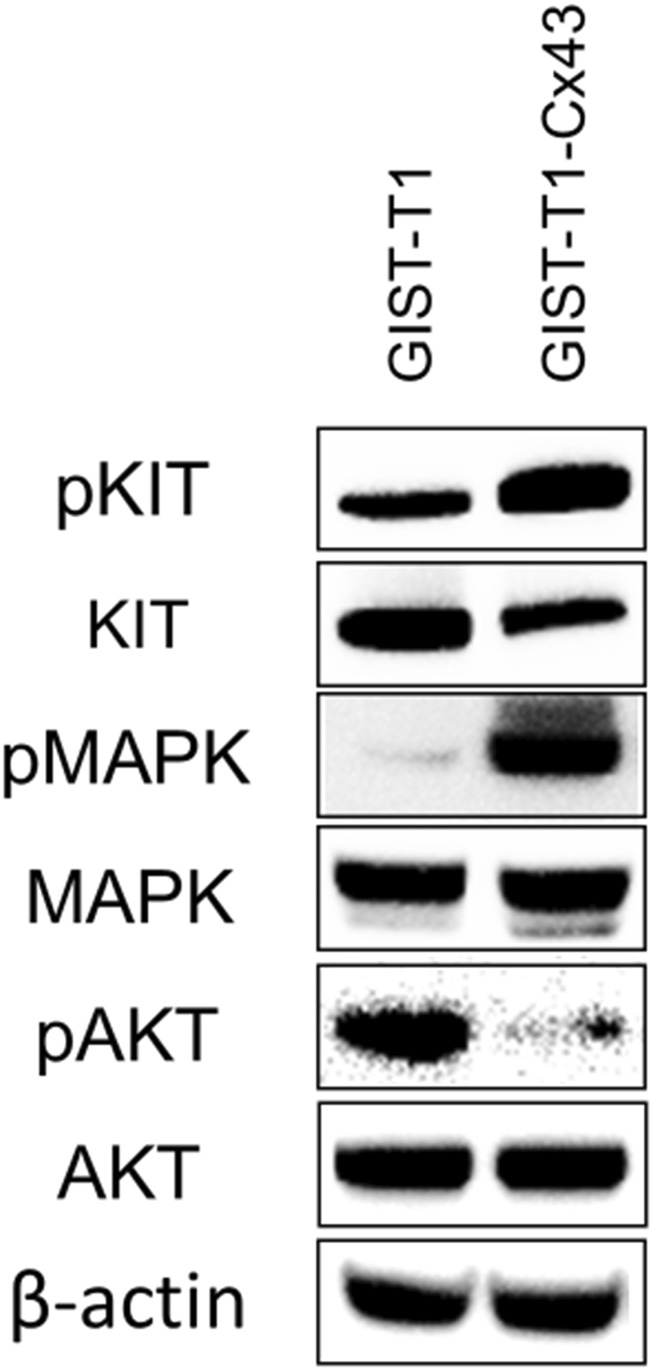
KIT signaling in GIST-T1 cells and GIST-T1-Cx43 cells. Western blot analysis revealed that MAPK phosphorylation was activated, whereas AKT phosphorylation was suppressed in the downstream molecules of the KIT signaling pathway in GIST-T1-Cx43 cells compared to that in GIST-T1 cells.

KIT expression, its phosphorylation, and the phosphorylation of downstream molecules of the KIT signaling pathways, including PI3K-AKT and RAS-RAF-MAPK, were examined. MAPK phosphorylation was activated, whereas AKT phosphorylation was suppressed in GIST-T1-Cx43 cells compared to that in the original GIST-T1 cells (Figure 7). The RAS-RAF-MAPK pathway was significantly activated in GIST-T1-Cx43 cells.

## Discussion

Patients diagnosed with SI-GISTs are considered to have a poorer prognosis than those with G-GISTs, primarily because of the elevated risk of metastasis and tumor-related mortality [14, 15, 17]. Our previous research demonstrated that Cx43 and cell adhesion molecule 1 (CADM1) are predominantly expressed in most SI-GISTs, whereas their expression is infrequent in G-GISTs [20, 33]. To investigate whether elevated *CADM1* expression in SI-GISTs influences the biological behavior of GISTs, we conducted *in vitro* experiments comparing original GIST-T1 cells, which exhibit very low *CADM1* expression, with GIST-T1 cells engineered to express high levels of *CADM1* (GIST-T1-CAD cells) [32]. The findings revealed that GIST-T1-CAD cells exhibited reduced capabilities for proliferation, migration, and invasion but demonstrated enhanced adhesion to and transmigration through the endothelium compared with original GIST-T1 cells [32]. Subsequently, we explored the impact of high *Cx43* expression in SI-GISTs on GIST biological dynamics. In this study, we successfully developed novel *in vivo* liver metastasis models through intrasplenic injection transplantation and corroborated conventional *in vitro* experiments by comparing original GIST-T1 cells with low *Cx43* expression and GIST-T1-Cx43 cells with high *Cx43* expression. The results indicated that GIST-T1-Cx43 cells possessed a greater propensity for metastasis, migration, invasion, adhesion, and transmigration through the endothelium than the original GIST-T1 cells, with the exception of the proliferation potential. Furthermore, the RAS-RAF-MAPK pathway was significantly activated in GIST-T1-Cx43 cells compared to that in the original GIST-T1 cells. The RAS-RAF-MAPK pathway is crucial for tissue remodeling and cell migration, both essential processes for tumor invasion and metastasis [34]. In cancer, the aberrant activation of this pathway often results in increased cell proliferation and resistance to apoptosis, thereby promoting tumor growth and metastasis [34]. These findings imply that *Cx43* may enhance the liver metastatic potential of GIST cells.

*Cx43* is the most ubiquitously expressed and extensively studied connexin in human tissues. It is a four-pass transmembrane protein responsible for the formation of gap junctions and hemichannels [22]. *Cx43* is implicated in various stages of tumor progression, from initiation to metastasis, with its expression varying according to the stage of tumor progression [35]. Recent studies have indicated that *Cx43* may be transiently upregulated during the later stages of metastasis in certain tumors [21, 22, 35]. It has been suggested that the aberrant expression of *Cx43* could play a crucial role in the peritoneal metastasis of gastric cancer cells, and that Cx43-mediated heterocellular gap-junctional intercellular communication between gastric cancer cells and mesothelial cells might constitute a significant regulatory step during metastasis, particularly during the transmigration of gastric cancer cells through the mesothelial cell barrier [36]. Furthermore, it has been demonstrated that the upregulation of *Cx43* enhanced the adhesiveness of breast cancer cells to pulmonary endothelial cells, suggesting that *Cx43* may contribute to metastatic tumorigenesis and tumor vasculogenesis [37]. These findings support the hypothesis that *Cx43* is significantly involved in the metastasis of cultured GIST cells. Notably, the dual role of *Cx43* in both tumor suppression and promotion of cell migration and invasion observed in this study parallels that seen in gliomas [38], suggesting that similar mechanisms may exist between GISTs and gliomas.

Elevated *Cx43* expression may indicate poor prognosis in patients with SI-GISTs because of its association with enhanced metastatic potential. An antibody targeting Cx43 has the potential to inhibit SI-GIST metastasis by suppressing cellular migration, invasion, tumor-endothelial cell adhesion, and transendothelial migration. Our recent findings suggest that an antibody-drug conjugate (ADC) targeting CADM1 exhibits a significant antitumor effect on GIST cells with high *CADM1* expression, which is characteristic of SI-GISTs, in both *in vitro* and *in vivo* models [39]. An ADC targeting Cx43 may inhibit GIST metastasis, and ADCs targeting both Cx43 and CADM1 may offer even greater efficacy.

Given the indolent nature of GISTs, establishing a model for liver metastasis has proven challenging, and no such model has been previously documented. In this study, we present findings from research on a GIST liver metastasis model, substantiated by *in vitro* validation experiments for the first time. However, this study had some limitations. First, we demonstrated the potential involvement of *Cx43* expression in the metastasis of SI-GISTs using only *in vivo* liver metastasis models. Considering that GISTs frequently result in liver metastasis and peritoneal dissemination, it is imperative to verify whether analogous results can be replicated *in vivo* using models of peritoneal dissemination. Second, we did not thoroughly investigate whether *Cx43* influences the expression of other surface adhesion molecules, such as *CADM1*, as previously reported [32, 33]. Other surface molecules, in addition to *Cx43*, may influence the migration, invasion, and interaction of GIST cells with HUVECs. Transfection of *Cx43* cDNA into GIST-T1 cells may also modify the expression of surface adhesion molecules and downstream signaling activity, warranting further investigation. Third, we focused on the association between *Cx43* and vascular endothelial cells in SI-GIST metastasis. It has been proposed that *Cx43* may regulate angiogenesis and immune cell evasion within the tumor microenvironment. Thus, the mechanisms underlying these effects in GIST cells require further investigation.

## Conclusion

In GISTs, *Cx43* may facilitate the progression of liver metastasis, as evidenced by its involvement in cell migration, invasion, and interactions with vascular endothelial cells. Elevated *Cx43* expression may correlate with poor prognosis in patients with SI-GISTs owing to its enhanced metastatic potential. Consequently, *Cx43* may be a viable therapeutic target for treating SI-GISTs, particularly for inhibiting metastasis.

## Data Availability

The datasets presented in this study can be found in online repositories. The names of the repository/repositories and accession number(s) can be found in the article/Supplementary Material.
