# Supplementary material for: Connexin 43 enhances liver metastatic ability of GIST cells in vivo
Source: Pathol Oncol Res. 2026 Jun 4;32:1612383. doi: 10.3389/pore.2026.1612383 (PMC13275379; doi:10.3389/pore.2026.1612383)
Supplement: Supplementary file 1 [file Table1.docx]

**Supplementary Table S1**

Forward primer (5′→3′)　 CCGGGATCCGCCATGGGTGACTGGAGCGCCTT

Reverse primer (5′→3′)　 CCGCTCGAGCTAGATCTCCAGGTCATCAG
