# Supplementary figures and images for: Connexin 43 enhances liver metastatic ability of GIST cells in vivo
Source: Pathol Oncol Res. 2026 Jun 4;32:1612383. doi: 10.3389/pore.2026.1612383 (PMC13275379; doi:10.3389/pore.2026.1612383)

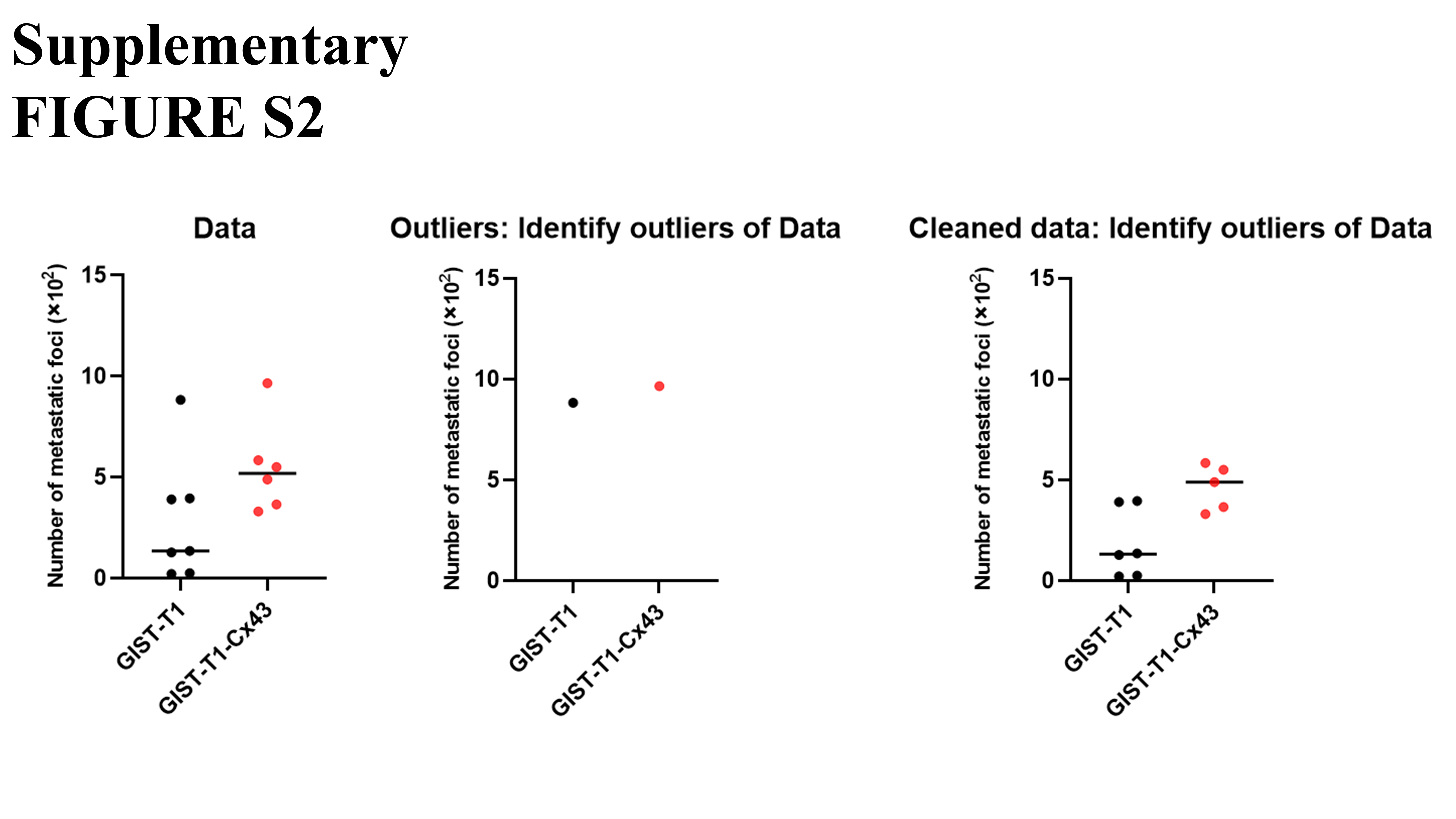

Supplement: Supplementary file 2 [file Image2.tif]

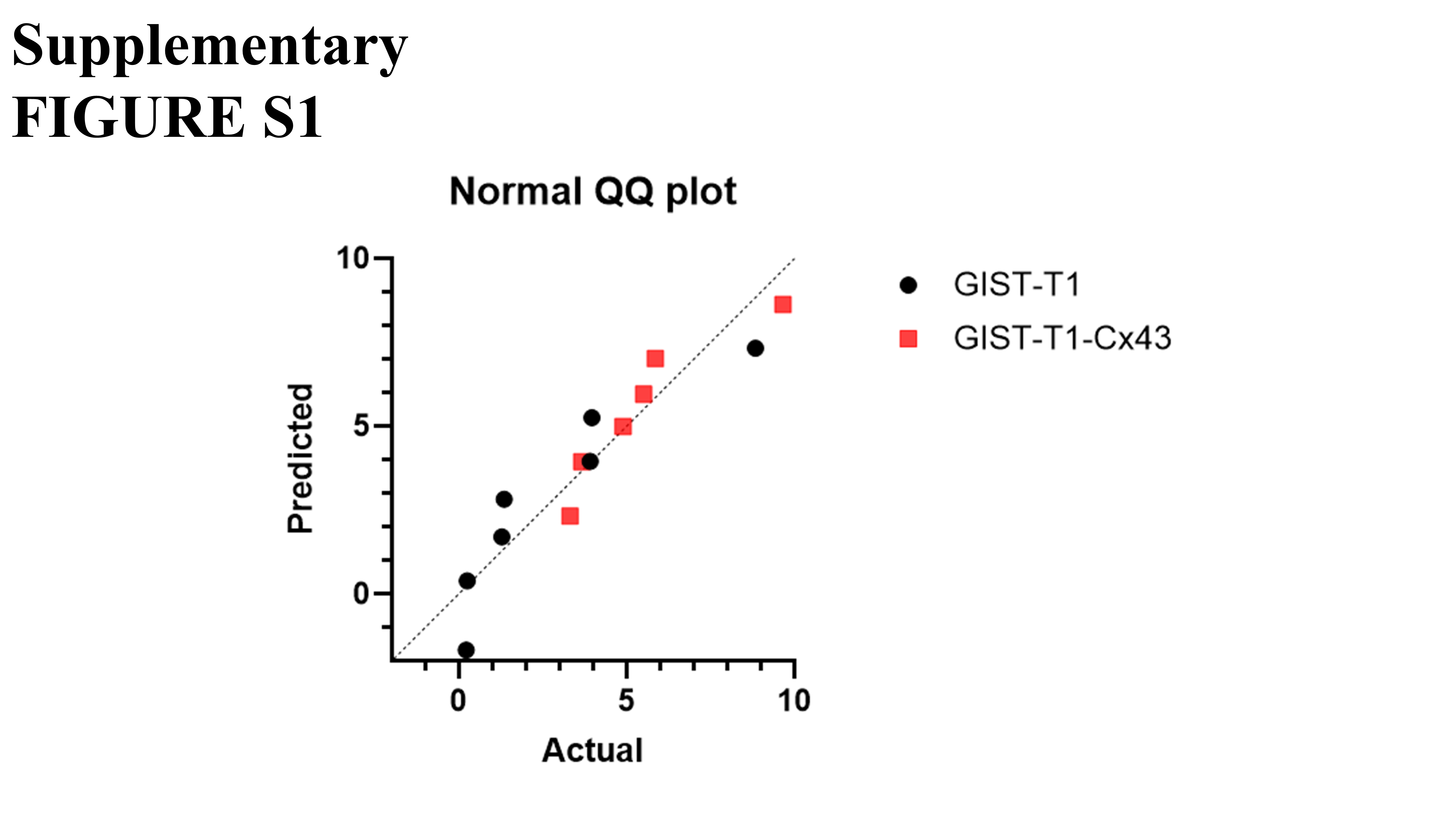

Supplement: Supplementary file 3 [file Image1.tif]
